# Morphological, genetic and epigenetic aspects of homoploid hybridization between *Salvia officinalis* L. and *Salvia fruticosa* Mill.

**DOI:** 10.1038/s41598-019-40080-0

**Published:** 2019-03-01

**Authors:** Ivan Radosavljević, Sandro Bogdanović, Ferhat Celep, Maja Filipović, Zlatko Satovic, Boštjan Surina, Zlatko Liber

**Affiliations:** 1 University of Zagreb, Faculty of Science, Department of Biology, Division of Botany, Marulićev trg 9A, HR, 10000 Zagreb, Croatia; 2 Centre of Excellence for Biodiversity and Molecular Plant Breeding (CroP-BioDiv), Svetošimunska cesta 25, HR, 10000 Zagreb, Croatia; 3 University of Zagreb, Faculty of Agriculture, Department of Agricultural Botany, Svetošimunska cesta 25, HR, 10000 Zagreb, Croatia; 4 Department of Biology, Faculty of Arts and Sciences, Kırıkkale University, Kırıkkale, Turkey; 5 University of Zagreb, Faculty of Agriculture, Department of Seed Science and Technology, Svetošimunska cesta 25, HR, 10000 Zagreb, Croatia; 6 Natural History Museum Rijeka, Lorenzov prolaz 1, HR, 51000 Rijeka, Croatia; 7 University of Primorska, Faculty of Mathematics, Natural Sciences and Information Technologies, Glagoljaška 8, SI, 6000 Koper, Slovenia

## Abstract

The inheritance of phenotypic, genetic and epigenetic traits in hybridization events is difficult to predict, as numerous evolutionary, ecological, and genetic factors can play a crucial role in the process of hybridization. In the middle Adriatic island of Vis, we investigated hybridization between *Salvia officinalis* and *S*. *fruticosa* at morphological, genetic and epigenetic levels. SSR results revealed that hybrid individuals were characterized by diploid set of chromosomes suggesting homoploid hybridization. A well-defined group that mostly comprised of F_1_ generation individuals was detected. For the majority of analysed morphological characteristics, hybrids were placed in-between parental taxa, while at the same time, values of different genetic parameters were mostly higher in hybrids than in parental species. The results revealed a high contrast in the levels of phenotypic variability and epigenetic excitation between parental taxa. Environmental niche modelling confirmed that in the studied location *S*. *officinalis* experiences optimal climatological conditions, while *S*. *fruticosa* struggles with unsuitable conditions. Very low levels of gene flow between the parental species were detected. In addition, contrasting levels of epigenetic excitation in the studied groups clearly demonstrated the importance of an epigenetic response to an altered environment and confirmed the trans-generational nature of the epigenetic changes.

## Introduction

Introduction of plant species into new areas occurs naturally, but the frequency of this phenomenon has increased dramatically because of human activity during the last few millennia. Consequently, hybridization between congeneric species occurs more often, causing significant genetic changes in both the native and alien species^1^. Hybridization is one of the fundamental forces of evolution, and this process can result in a diverse range of outcomes, including hybrid divergence and sympatric speciation, introgression, hybrid zone formation, erosion of species boundaries and possible species extinction through assimilation^2,3^. New lineages of hybrid origin are characterized either by allopolyploidy, meaning that the complete genomes of the parental species have joined, or homoploidy. As defined by Nieto Feliner *et al*.^4^, homoploid hybrid speciation “is the formation of a new-hybrid-species, independent from its parents, via hybridization with no whole-genome duplication and thus no increase in ploidy”. Although this definition seems to be straightforward, key aspects of hybridization process are under debate^4,5^. Schumer *et al*.^5^ proposed following criteria that must be met to confirm inter-species homoploid hybridization: reproductive isolation of hybrids must be recognized, there must be evidences that this isolation was triggered by hybridization itself and hybridization must be confirmed at a genomic level. However, in a recently published paper, Nieto Feliner *et al*.^4^ argues these criteria, especially the importance of reproductive isolation as a crucial criterion as an evidence of occurrence of a homoploid hybrid speciation.

Once an organism finds itself in an altered environment, its genotype can produce numerous phenotypes, of which some will presumably be better adapted to the new ecological conditions. This phenomenon, known as phenotypic plasticity, has an integral role in an organism’s attempt to cope with environmental variation and enables plants to respond to such variations within their lifetime^6,7^. Such expression of different phenotypes in changing environments may be caused by alteration of gene expression without changes in the primary sequence of the DNA, i.e., epigenetics^8,9^. Molecular mechanisms that are considered responsible for modifications in gene expression include histone and chromatin modifications and methylation, all of which are directed by small RNAs^10^. Thanks to transgenerational inheritance of epigenetic variants, phenotypic responses to an altered environment remain memorized and at disposal for generations to come^11^. Interspecies hybridization triggers not only genetic but also epigenetic changes through modification of DNA methylation patterns^12,13^. Consequently, new phenotypic variants found in hybrid offspring more often originate from epigenetic than genetic recombination, and thanks to their transgenerational heritability, they can play a very important role in the process of speciation^11,14,15^.

With approximately 1000 recorded species, the genus *Salvia* is the largest genus of the Lamiaceae family^16^. Thirty-six taxa are present in Europe, and these taxa are divided into seven sections^17^. The *Salvia* section includes 13 species, with the common or Dalmatian sage (*Salvia officinalis* L.) as its type species. Another easily recognizable member of this section is the Greek sage (*S*. *fruticosa* Mill.). Both species are well known due to their high essential oil content and commercial value^18–20^. The natural distribution range of common sage includes the eastern Adriatic coast from the Gulf of Trieste in the north to northwestern Greece in the south^21,22^, as well as parts of the central and southern Apennines^23–25^. Greek sage extends from Cyrenaica, Sicily, and southern Italy through the southern part of the Balkan Peninsula to West Syria^21,26^. Because both species have been highly prized for their numerous beneficial healing properties, Phoenicians, Greeks and Romans have introduced these species into many Mediterranean countries throughout history^26^. Ancient Mediterranean cultures were prone to transfer and grow useful plants near their settlements and colonies scattered across the Mediterranean basin. Consequently, sub-spontaneous populations and dubiously native populations of certain species can today be found in different locations where the ancient colonies once settled^27–29^.

In the central Adriatic region, a small and isolated population of Greek sage can be found on the island of Vis (Croatia)^30^, where the plant grows sympatrically with common sage. Because of the well-organized Greek colony Issa that was founded on the island of Vis in the 4^th^ century BC^31–33^, it can be assumed that this disjunct population of Greek sage located approximately 500 km from the nearest population of its type is of sub-spontaneous origin rather than being a member of an indigenous flora.

Hybridization within the section *Salvia* has been previously recorded (*S*. *lavandulifolia* Vahl. subsp. *vellerea* × *S*. *officinalis*^34^, *S*. *officinalis* × *S*. *lavandulifolia* subsp. *lavandulifolia*^35^, *S*. *tomentosa* Mill. × *S*. *officinalis*^18^); thus, it appears that erosion of species boundaries between closely related species of the section is present. Hybridization between common sage and Greek sage has never been documented to occur spontaneously in nature but was artificially performed for breeding purposes in Israel^18,19,36^. An obtained commercial sage hybrid (*Salvia officinalis* × *Salvia fruticosa* cv. *Newe Ya’ar No*. *4*) was well suited for intensive agricultural conditions in Israel, but in terms of essential oil quality, it was more similar to common sage than Greek sage^19^.

Although the distributions of both species overlap in parts of southern Albania and northern Greece^26^, hybridization between these species in the region seems unlikely because of the different flowering periods^37^. However, in the western coastal area of the Island of Vis, common and Greek sage have overlapping flowering periods from late April to early May, and hybridization between the two species is frequent, as a full spectrum of intermediate individuals sharing morphological traits of both parental taxa can be observed. Greek sage and hybrids grow in dense and admixed stands, whereas common sage is present sporadically. However, in the surrounding area, common sage grows abundantly as a typical representative of the indigenous flora. In addition, it is important to note that both species share the same number of chromosomes (2n = 14)^26^ and rely on the same pollinator assemblage^38,39^.

Because of their high variability, codominant inheritance and high reproducibility and reliability, for the last two decades microsatellites have been one of the most popular molecular markers used in many different areas, from genome mapping^40,41^ to population and conservation genetics^42^. However, unlike universal molecular markers (e.g. AFLP^43^) that require no prior knowledge regarding target species genome, microsatellites are species-specific and require *de novo* isolation for any species being studied for the first time^44^. Also, in situations of weak population differentiation, an analysis based on limited number of microsatellite loci lacks the statistical power for individual population assignment^45^. Since this limitation can be overcome only by significant increase of the number of used loci^46^ which may be technically challenging and time consuming^45^, the utilization of AFLP technique (Amplified fragment length polymorphism) in such situations represents a good alternative^45^. However, AFLPs are also characterized by some major drawbacks when compared to microsatellites. The most important ones are homoplasy^47^ and their dominant nature^45^. While the homoplasy (i.e. either non-homologous fragments share the same or very similar size or independent fragment loss occur) decreases the resolution and reliability of the analysis^47^, AFLPs dominancy makes it impossible to assess the levels of departure from Hardy-Weinberg equilibrium^45^. In summary, microsatellites and AFLPs both have certain advantages against each other that should be weighted when choosing the most appropriate genotyping technique for a specific study. The MSAP technique^48^ (Methylation sensitive amplification polymorphism) as the most widely used tool in epigenetic studies of natural populations^49^, is a modification of the AFLP method. In the majority of MSAP based studies, the rare cutter *EcoR*I restriction enzyme was used as in the AFLP analysis, while the frequent cutter *Mse*I was replaced by *Hpa*II and *Msp*I methylation-sensitive restriction enzymes^50^. Since these two methods are technically very similar and their results can be easily compared to each other, they are methods of choice in studies of both genetic and epigenetic diversity of the same target species.

The main goal of this study was to perform a comprehensive analysis of hybridization between common sage and Greek sage at morphological, genetic and epigenetic levels. For this purpose, in sampled individuals, we analysed 39 morphometric traits and performed DNA analysis using microsatellite and AFLP nuclear genetic markers as well as MSAP epigenetic markers. By exploring the different aspects of the hybridization event, we aimed to (1) obtain insight into the extent of hybridization between common sage and Greek sage on a phenotypic level; (2) determine the prevalence of hybridization and assess the nature and levels of inter-species gene flow (if present); (3) assess the levels of methylation variability in hybrids and parental groups; and (4) discuss the complexity of the interactions among the environment, phenotype, genotype and epigenotype through the prism of core vs. marginal population.

## Results

### Morphological diversity

The mean Shannon’s diversity index based on 23 qualitative traits was significantly different among taxa (P < 0.05), with means (calculated from all traits) of 0.155, 0.417 and 0.684 for common sage, hybrids and Greek sage, respectively (Supplementary Table S1). The number of monomorphic traits was the highest in common sage (17), the lowest in Greek sage (one) and intermediate in the group of hybrids (nine). Fourteen of the 23 qualitative traits were the most variable in Greek sage and six in the group of hybrids, whereas only two traits were the most variable in common sage. A single trait (Trichome type: Eglandular, Subadpressed) that was monomorphic for all analysed individuals, was excluded from further analysis. For common sage, 14 out of 15 individuals had the same specific trichome pattern, whereas only two pairs out of 34 individuals had the same pattern for Greek sage. Each of the remaining 32 individuals exhibited a unique trichome pattern. Among 26 hybrid individuals, 11 specific trichome patterns were observed, eight of which could not be found in either parental species. For common sage, only three different trichome types were identified; for the other two groups, six different types were identified (Supplementary Table S2). Analysis of variance (ANOVA) indicated significant differences in all analysed quantitative traits. The mean values of the most traits in the hybrid specimens were intermediate between the mean values of the two parental taxa (Supplementary Table S3). The correlation between Gower’s distance matrices based on qualitative and quantitative traits was highly significant (r = 0.41; P < 0.0001).

The mean intraspecies Gower’s distance was the highest in Greek sage and lowest in common sage, while an intermediate value was detected in the hybrids (0.274, 0.097 and 0.239, respectively). All pairwise differences in mean intraspecies Gower’s distances were significant at P < 0.05.

**Figure 1:**
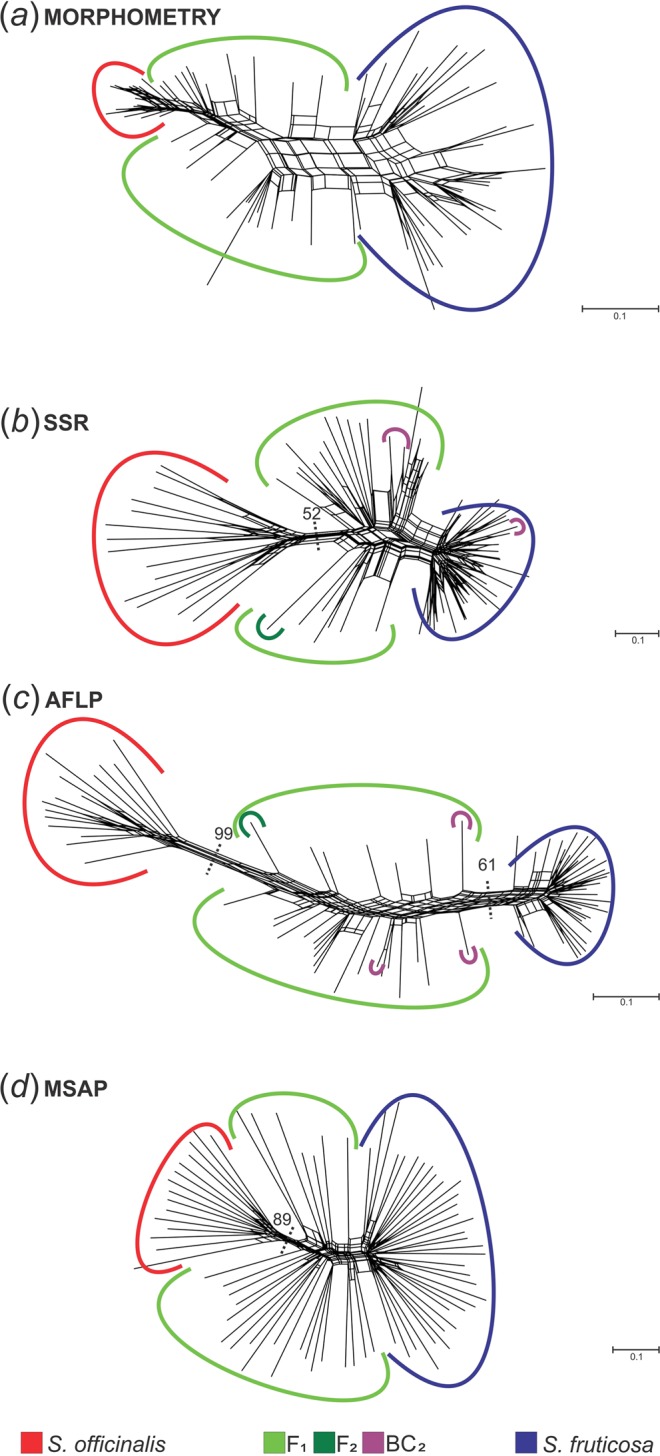
Neighbour-net diagrams showing relationships among studied individuals based on results from different analysis methods. Diagrams are based on (**a**) Gower’s distances of 22 qualitative and 16 quantitative morphological characters for a morphometry analysis, (**b**) a proportion-of-shared-alleles distance matrix for SSR analysis, (**c**) a Dice distance matrix for AFLP analysis and (**d**) a Dice distance matrix for MSAP analysis. In both neighbour-net diagrams based on SSR and AFLP results, affiliation of individuals to different classes was based on the NewHybrids results from a microsatellite dataset. Bootstrap support values were derived from Neighbour Joining analysis, and those greater than 50% are indicated.

A neighbour-net diagram based on Gower’s distance matrices of combined qualitative and quantitative traits unambiguously separated the parental taxa from each other and from the hybrids (Fig. 1). The hybrids were positioned between the parental taxa.

### SSR diversity

All seven microsatellite loci were successfully amplified in all samples. For each individual, analysis revealed either one (i.e. homozygous state) or two (i.e. heterozygous state) allele per locus. Overall, 64 alleles were detected, out of which 48 were present in common sage, 24 in Greek sage and 52 in hybrids, with a considerable number of alleles shared between taxa (Supplementary Fig. S1). Private alleles were detected in common sage (10) and in the hybrids (five), but not in the Greek sage. Consequently, common sage had the highest level of private allelic richness, the hybrids had an intermediate level, and Greek sage had the lowest level (2.00, 0.52 and 0.13, respectively).

Further comparison across the studied taxa indicated significant differences in patterns of genetic variation. The levels of observed (*H*_*O*_) and expected heterozygosity (*H*_*E*_) were significantly higher in common sage (0.610 and 0.749, respectively) and in the hybrids (0.778 and 0.678, respectively) than those in Greek sage (0.289 and 0.333, respectively). Significant and positive *F*_*IS*_ values were detected in both parental species (0.187 and 0.132 in common sage and Greek sage, respectively), whereas hybrids exhibited a negative but not significant *F*_*IS*_ value of −0.147 (Supplementary Table S4).

The neighbour-net analysis (Fig. 1) clearly separated the parental taxa from the hybrids. The highest levels of within-taxon genetic distances were observed in common sage, followed by the hybrids and then by Greek sage (0.684, 0.522 and 0.289, respectively). Expectedly, the highest average genetic distance was detected between the parental species (0.876) (Supplementary Table S5).

Although the AMOVA indicated that 77% of the genetic variation was observed within the groups (Supplementary Table S6), the highly significant *ϕ*_*ST*_ values confirmed the genetic differentiation among the taxa (Supplementary Table S5).

As expected, the highest pairwise *ϕ*_*ST*_ was noted between the parental species (0.431), while the value was lowest between the hybrids and Greek sage (0.115).

**Figure 2:**
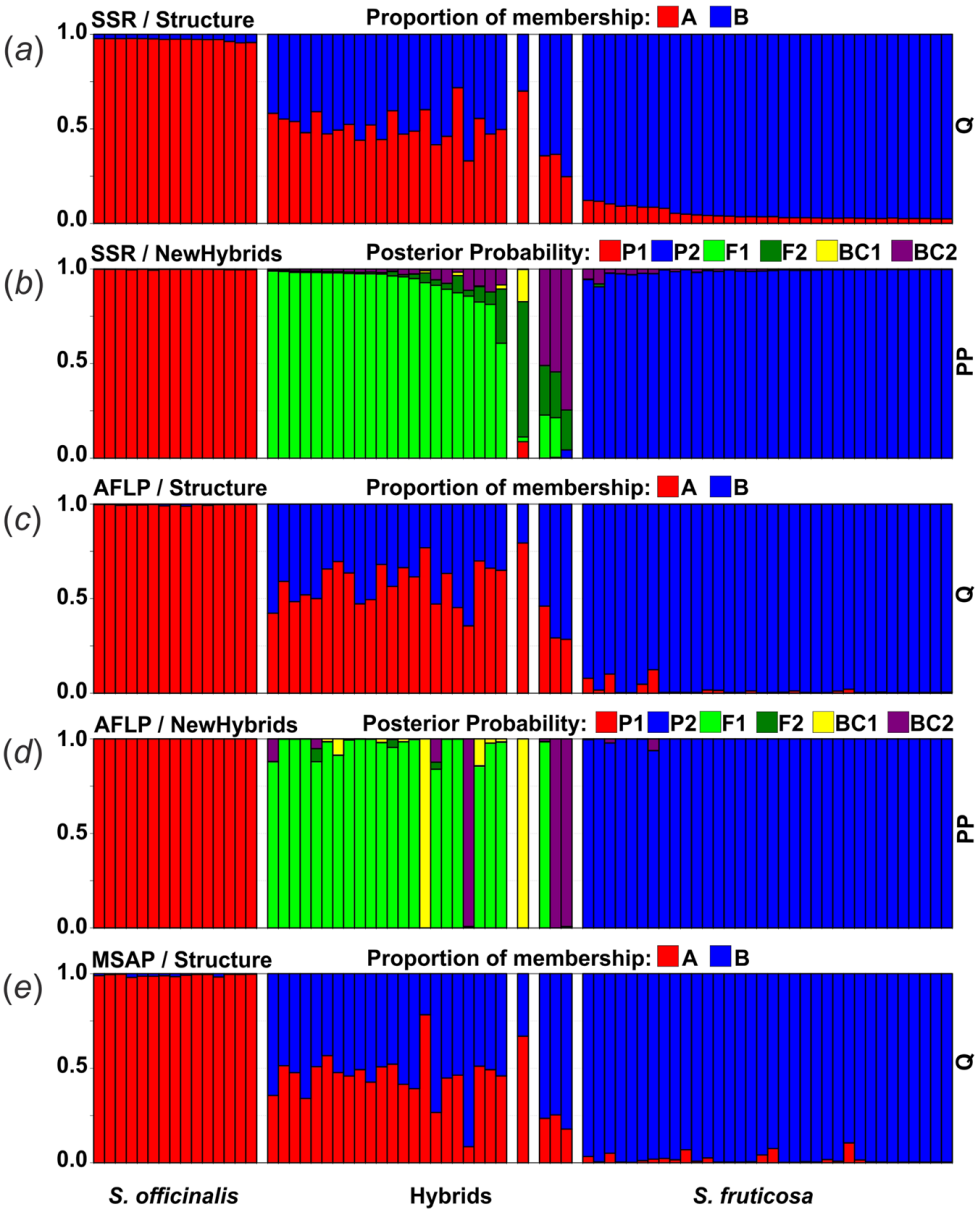
Genetic structure and assignment of individuals into classes as assessed by the computer programs STRUCTURE and NEWHYBRIDS. Each individual plant is represented by a single vertical line. For STRUCTURE (**a**,**c**,**e**), each colour (red and blue) represents a cluster (*S*. *officinalis* and *S*. *fruticosa*, respectively), and the length of the coloured segments indicates the individual’s estimated proportions of membership in those clusters. For NEWHYBRIDS (**b**,**d**), each colour (red, blue, light green, dark green, yellow and violet) represents a genealogical class (purebred *S*. *officinalis*, purebred *S*. *fruticosa*, F_1_ generation, F_2_ generation, back-cross to *S*. *officinalis* and back-cross to *S*. *fruticosa*, respectively). The length of the coloured segments indicates the individual’s estimated posterior probability of assignment to each class.

The results from the Bayesian analysis implemented in STRUCTURE strongly supported the presence of two clusters representing the parental species (Fig. 2). In individuals *a priori* identified as hybrids, wide range of admixture proportions were observed. The average estimates of the likelihood of the data, conditional on a given number of clusters, *ln*[Pr(X|K)], increased up to K = 3, with a standard deviation substantially higher at K = 3 compared to that at K = 2. The highest ΔK was obtained at K = 2 (177.54), and the second highest ΔK was obtained at K = 3 (4.41).

The assignment of hybrids using NEWHYBRIDS was congruent with the results of STRUCTURE; all individuals characterized by admixed proportions were confirmed to be of a hybrid origin (Fig. 2). The majority of the hybrid individuals (22 out of 26) were classified as F_1_ hybrids with posterior probabilities (PP) ranging from 0.608 to 0.990. A single hybrid individual was classified as an F_2_ (PP = 0.715), and three individuals were classified as back-crosses to Greek sage (BC_2_) (PP = 0.510, 0.544 and 0.745). All common sage and Greek sage samples were classified as pure parental (P_1_ and P_2_, respectively) with posterior probabilities ranging from 0.994 to 0.999 and 0.906 to 0.998, respectively. Assignment of individuals into classes (parental: *S*. *officinalis* and *S*. *fruticosa*, hybrid: F_1_, F_2_ and BC_2_) carried out using NEWHYBRIDS was also indicated in the neighbour-net diagrams (Fig. 1).

### AFLP diversity

The estimated error rate per primer combination in the AFLP analysis ranged from 0.68% to 2.9%. The mean error rate was 1.94%, which is similar to the error rate found in other AFLP studies^51^. The highest proportion of detected polymorphic loci was observed in the group of hybrids, followed by the Greek sage and common sage (0.739, 0.582 and 0.455, respectively). According to Shannon’s information index, a significantly (P < 0.05) higher level of diversity was detected in the group of hybrids as compared to that of the parental groups, while no significant difference in levels of genetic diversity was observed between the parental groups (Supplementary Table S7).

Average Dice’s genetic distance at the taxon level was significantly higher (P < 0.05) in both common sage and the hybrids than that in the Greek sage (0.297, 0.281 and 0.178, respectively), while at the inter-taxa level the greatest genetic distance was expectedly observed between the parental taxa (0.836), while the least was between the Greek sage and the hybrids (0.421) (Supplementary Table S5). Neighbour-net analysis supported the obtained results (Fig. 1). Analysis of molecular variance showed that the majority (57%) of total genetic variability can be attributed to variability detected among groups (Supplementary Table S6), while highly significant pairwise *ϕ*_*ST*_ values between the involved taxa supported the existence of three differentiated groups of individuals (Supplementary Table S5). Similar to the results of microsatellite analysis, the highest pairwise *ϕ*_*ST*_ value was detected between the parental taxa (0.733).

We have chosen a NEWHYBRIDS result based on SSR analysis as a referent result in terms of assigning individuals to different classes (i.e., parental species, F_1_ generation, F_2_ generation and back-crosses to either of the parental species) in the AFLP and MSAP analyses. The results from STRUCTURE and NEWHYBRIDS based on the AFLP dataset strongly supported the results obtained by microsatellite analysis (Fig. 2).

The average estimates of the likelihood of the data, conditional on a given number of clusters, *ln*[Pr(X|K)], increased up to K = 3, with the standard deviation substantially higher at K = 3 compared to K = 2. The highest ΔK was obtained at K = 2 (3204.10), and the second highest ΔK was obtained at K = 3 (609.19).

In the STRUCTURE results, two parental clusters were well recognized, while in the group of hybrids, a wide range of admixture proportions can be observed. The results obtained by NEWHYBRIDS were for the most part congruent with the NEWHYBRIDS results for the microsatellites, with the exception of three individuals within the group of hybrids. Two individuals recognized as F_1_ hybrids in the SSRs result were now recognized as a back-cross to common sage (BC_1_) and a back-cross to Greek sage (BC_2_). The individual classified as an F_2_ hybrid in the SSRs result was now recognized as a back-cross to common sage (BC_1_).

### MSAP diversity

**Figure 3:**
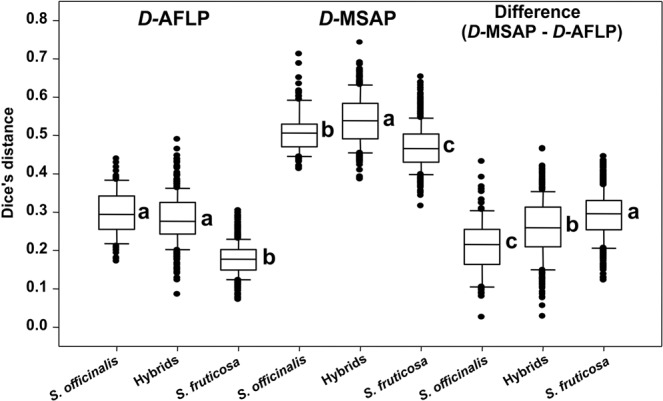
Box plots of intraspecies distances based on AFLP and MSAP data by species.

Analysis of MSAP data revealed that the highest proportion of polymorphic loci could be observed in the group of hybrids, followed by Greek sage and common sage (0.731, 0.715 and 0.545 respectively), as was also the case with the AFLP results (Supplementary Table S7). Additionally, Shannon’s information index suggested the same ration of diversity levels among studied groups as that seen in the AFLP results: parental groups were characterized by significantly lower levels of epigenetic diversity (0.325 for common sage and 0.328 for Greek sage) than was the group of hybrids (0.367), while there was no significant difference between the diversity levels of the parental groups (Supplementary Table S7). Dice distance at an intra-taxa level was significantly lower (P < 0.05) in Greek sage (0.470) than in common sage (0.508), while in the group of hybrids (0.540) it was significantly higher than that seen in both the parental species (Supplementary Table S5). However, when comparing values of Dice distances from the MSAP and AFLP results for each individual group, the detected difference was significantly higher (P < 0.05) in the Greek sage (0.292) then it was in the group of hybrids (0.258), while the latter was significantly higher than that of common sage (0.212) (Fig. 3).

The results for Dice distance between researched groups in the MSAP analysis were fully congruent with genetic distance results from the AFLP data analysis, as the greatest distance was detected between the parental taxa (0.717) and the least distance between Greek sage and the hybrids (0.565) (Supplementary Table S5). These results were also well supported by neighbour-net analysis (Fig. 1).

In contrast to the AFLP results, AMOVA analysis based on the MSAP dataset revealed that the majority (81%) of the total genetic variability was attributed to variability detected within groups, rather than among them (Supplementary Table S6). Pairwise *ϕ*_*ST*_ values between the involved taxa were again highly significant but of smaller amplitude than those in the AFLP results. (Supplementary Table S5).

The average estimates of the likelihood of the data, conditional on a given number of clusters, *ln*[Pr(X|K)], increased up to K = 3, with a standard deviation substantially higher at K = 3 compared to K = 2. The highest ΔK was obtained at K = 2 (1778.52), and the second highest ΔK was obtained at K = 3 (664.64).

Results obtained by STRUCTURE for the MSAP dataset were consistent with STRUCTURE results for microsatellite and AFLP analysis as the parental species were well recognized. All individuals belonging to different generations of hybrids as defined by NEWHYBRIDS analysis based on microsatellites were again characterized by a range of admixture proportions (Fig. 2).

### Environmental-niche modelling

**Figure 4:**
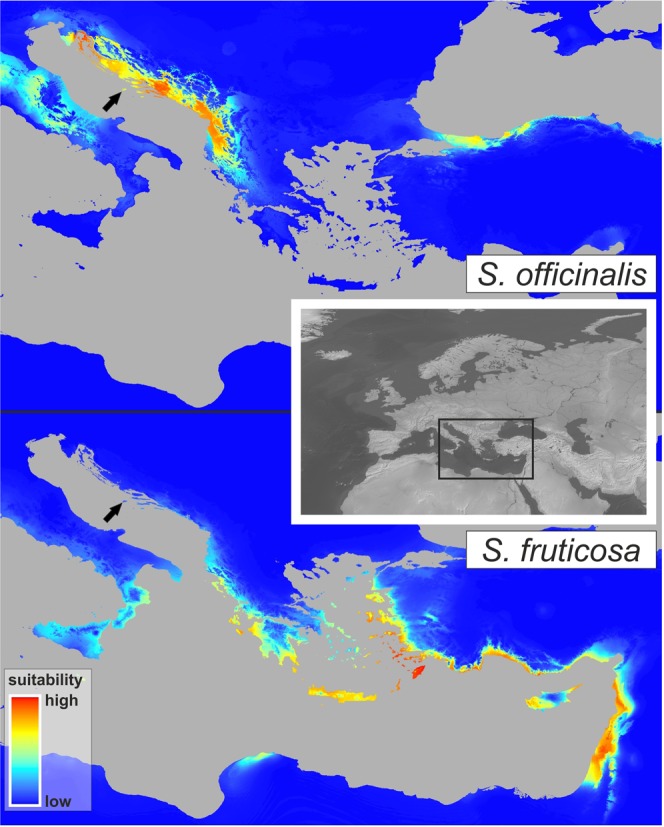
Environmental suitability map for *S*. *officinalis* and *S*. *fruticosa* based on ecological-niche modelling. The satellite imagery was obtained from Natural Earth public domain map dataset (https://www.naturalearthdata.com/downloads/10m-raster-data/10m-gray-earth/). Environmental suitability maps were generated from the climate layers obtained from http://www.worldclim.org113 and modified using ArcGIS ver. 10.1. (Esri, Redlands, CA, USA). The final figure was constructed using CorelDraw Graphics Suite X7 (Corel Corp., Ottawa, Canada).

Based on the multicollinearity test, 12 bioclimatic variables for *S*. *officinalis* (i.e. BIO1, BIO2, BIO5, BIO6, BIO7, BIO11, BIO12, BIO15, BIO16, BIO17, BIO18 and BIO19) and 13 for *S*. *fruticosa* (i.e. BIO1, BIO2, BIO5, BIO6, BIO7, BIO10, BIO11, BIO13, BIO14, BIO16,BIO17, BIO18 and BIO19) were excluded from further analysis. For both species, models with the smallest AICc values were selected. All bioclimatic variables and relative contributions to the Maxent model of the selected variables are listed in Supplementary Table S8. Yielded models are well congruent with the common sage distribution area in the western parts of the Balkan Peninsula and the Greek sage distribution area in the central and eastern Mediterranean (Fig. 4). Moran’s I correlograms revealed a significant autocorrelation of ENM residuals for distances smaller than 150 km (for *S*. *officinalis*) or 250 km (for *S*. *fruticosa*). However, since both species are characterized by relatively small distribution areas that extends in narrow coastal areas, such result was inevitable, because reduction of number of occurrences to avoid ENM residual autocorrelation would lead to insufficiently large data set for this analysis. Besides, this result have not any influence on comparison of two ENMs which illustrates strong spatial segregation of *S*. *officinalis* and *S*. *fruticosa* environmental niches and positioning of hybrid population examined in this paper 1) far away from the environmental niche of *S*. *fruticosa* and 2) within the environmental niche of *S*. *officinalis*.

## Discussion

### Morphological aspects of inter-species hybridization

Analyses of morphological variation resulted in a clear delimitation of studied groups. Common sage individuals were characterized by nearly complete morphological uniformity, while in Greek sage, significantly higher levels of phenotypic variation were found (Fig. 1, Supplementary Table S3). Such contrasting results could likely be explained by the fact that the population of common sage is native to the Island of Vis and is positioned very close to the central part of the species’ distribution area. Additionally, the Island of Vis was a part of a larger refugium area where the species likely survived Quaternary climatic oscillations^52,53^. The long persistence of the species in this region led to its adaptation to local ecological conditions and resulted in it reaching a phenotypic optimum. On the other hand, the population of Greek sage is far away from the Eastern Mediterranean where it grows abundantly^26^. According to MaxEnt analysis (Fig. 4), the studied Greek sage population is faced with marginal environmental conditions that likely caused the high levels of phenotypic variation known as phenotypic plasticity^7,54,55^. It is known that when a population is faced with altered environmental conditions, a full spectrum of phenotypes is produced^56^. Over time, only those phenotypes with the highest fitness and best-fitted traits will be chosen by natural selection^57^.

As expected, hybrid plants were mostly characterized by admixed and/or intermediate morphological traits, as well as by unique traits that could not be identified in either of the parental species were found i.e., bract length (BL) and calyx lobe length (CLMI) were significantly longer in the hybrids than in either of the parental taxa (Supplementary Table S3). The occurrence of hybrid-specific characters was also confirmed by other studies^58^. The emergence of such novel and unpredictable traits may be related to numerous mechanisms, such as the combination and complementary action of parental alleles^58^, epistasis^59^, phenotypic plasticity^58^ and combinations of these phenomena.

### Multilocus genetic characterization of hybridization

Analysis of seven microsatellite loci revealed that the common sage group was characterized by significantly higher levels of genetic diversity than those of the Greek sage group (Supplementary Table S4). Such a result might be explained by specific evolutionary histories and past demographic fluctuations of these populations. Although today the Island of Vis is located 45 km from the mainland, during the Last Glacial Maximum (ca. 20,000 years ago), when sea level was 120 metres lower than it is today^60,61^, the island was part of the mainland. It seems that the last several thousand years of isolation was not enough for genetic depauperation of the large common sage population on the Island of Vis. In high contrast to the common sage results, the obtained microsatellite results for Greek sage strongly support assumption of its non-native origin in this area^62^. If we assume that the population originated from very few individuals (i.e., the founder effect) ca. 2500 years ago when Ancient Greeks started their colony on the island^31–33^ and consider that even today the population is comprised of a limited number of individuals, the detected levels of genetic variability are not surprising.

In the group of hybrids, the highest levels of genetic diversity and allelic richness were observed (Supplementary Table S4**)**. Common sage is characterized by greater genetic variability and participates in a hybridization event with more alleles than that of Greek sage, resulting in a high similarity of allelic richness levels between the hybrids and common sage. As already mentioned, only three out of the 26 hybrids were identified as backcrosses to Greek sage. Although such a result implicates that the gene flow is oriented towards the Greek sage as a sink population, only three detected BC_2_ individuals make such a conclusion speculative. However, such orientation of the gene flow would be in accordance with a neutral model which suggests that in a hybridization between the native and non-native species, the gene flow is expected to be biased towards the non-native species^63^. In addition, the model proposes that the asymmetry of the inter-species gene flow arises because of “a demographic imbalance between the two species at the wave front, where the invading species is at lower densities^63^”, thus supporting our assumptions regarding the non-native origin of the Greek sage in the studied location. Similar patterns of introgression were also detected by some other researches^64,65^. Based on obtained results it is possible to discuss the nature of homoploid hybridization in accordance to Schumer *et al*.^5^. The case study here presented obviously meet only one criterion without any doubts, as the genome level hybridization was confirmed. The criterion implying the necessity of reproduction isolation of hybrids from parental species was not met, although such a small number of back-crosses leaves some doubts. Finally, even if the back-crosses were not detected, based on implemented research strategies it would be impossible to address the issue of the origin of the reproduction isolation.

### Epigenetic analysis of the hybridization event: AFLP vs. MSAP

For an additional reliable assessment of genome-wide genetic and epigenetic variability in the hybridization event, technically very similar AFLP and MSAP approaches were used. In AFLP analysis there was no significant difference in Shannon’s information indexes between the parental taxa, which was not the case with results obtained by microsatellites. As already suggested, it is possible that such results were obtained as a consequence of cross-amplification of microsatellites in the target species (i.e., Greek sage). Based on both the AFLP and MSAP results, three groups of individuals clearly differentiated from each other. The results from STRUCTURE for both AFLP and MSAP and from NEWHYBRIDS for AFLP (Fig. 2) are fully congruent with NEWHYBRIDS results for SSRs. The only differences were in the positioning of the three individuals when comparing the SSR and AFLP results and one individual when comparing the SSR and MSAP results. Because these individuals were all recognized as hybrids but of different generation, such small differences in results are likely not of major significance and can be attributed to the noise of estimation.

In both the AFLP and MSAP results, the proportions of polymorphic alleles and Shannon’s information index have significantly (P < 0.05) higher values for the group of hybrids than those of the parental species, and such results are not surprising, as similar results were observed in other research^66^. When comparing differences between genetic and epigenetic distances (Fig. 3) within individual taxa, contrasting results occurred. The smallest difference was observed for common sage, intermediate for the hybrids and most pronounced for Greek sage. Such a result supports the assumption that the common sage population is well adapted to local environmental conditions and is consequently exposed to low levels of stress. At the same time, a very pronounced difference between the levels of genetic and epigenetic diversity in the Greek sage group most likely occurs because of the stressful environmental conditions encountered outside of the species’ optimal ecological niche (Fig. 4). Such results clearly confirm the strong influence of unfavourable environmental conditions on epigenetic excitation in the plants^9,67,68^.

Being characterized by intermediate values of difference between genetic and epigenetic diversity of parental species, the results for the group of hybrids support our knowledge of heritability of epigenetic traits^11,69–71^. The obtained results are well supported and visualized by the Neighbour-Net diagram (Fig. 1).

Analysis of molecular variance of the partitioning of AFLP diversity between and within studied groups revealed that 57.2% of the overall genetic diversity can be attributed to diversity among groups. Bearing in mind that the analysed hybridization event was at an inter-species level, such a result is expected. However, for epigenetic diversity, a highly contrasting result was obtained, as only 18.7% of the overall genetic diversity can be attributed to diversity among groups. Such results strongly support the idea that the majority of epigenetic changes are specific for each individual and in addition, that the epigenetic variability is not tightly dependent on an individual’s genetic diversity and structure^66,72,73^.

## Conclusions

In our research, different morphometric and molecular methods were used to analyse hybridization between two closely related yet mostly allopatric species. By doing this, we gained information on the different aspects of inheritance of phenotypic, genetic and epigenetic traits in contrast to different levels of adaptation of the parental populations to local environmental conditions. The obtained results revealed a high contrast between the levels of genetic and phenotypic variability in the parental taxa, as common sage was characterized by high levels of genetic and extremely low levels of phenotypic variability. For Greek sage, morphometric analysis revealed exceptionally high levels of variability that can likely be attributed to phenotypic plasticity, while results for genetic variability obtained using two methods were not entirely congruent with each other. Such results for a parental species can likely be explained by their different demographic histories in the studied location. A well defined group of hybrids was detected at morphological, genetic and epigenetic levels and were mostly comprised of F_1_ generation individuals, suggesting that the hybrids were predominantly sterile. For most analysed morphological characteristics, hybrids were placed in-between the parental taxa, while at the same time, their values of different genetic parameters were mostly higher than those of the parental species. In addition, epigenetic analysis of the involved taxa improved our understanding of the importance of epigenetic variation in natural populations in contrast to altered environmental conditions and confirmed the trans-generational nature of epigenetic changes. Although the obtained results provided substantial insight into the hybridization between closely related native and non-native plant species, additional knowledge would be gained by more comprehensive sampling of higher resolution accompanied by geocoding of each sample and genetic analysis of chloroplast DNA. By performing the spatial analysis of both inter-species gene flow and fertilization direction, further unravelling of the complexity of the hybridization event would be achieved.

## Methods

### Sampling of plant material

Leaf tissue and branches with inflorescences from 75 *Salvia* individuals were collected in the western part of the Island of Vis (Croatia) in late April. The morphological characteristic used in the field for identification of parental species and hybrids was the calyx shape (Supplementary Fig. S2).

The common sage calyx is larger and strictly bilabiated, whereas the calyx of Greek sage is smaller, tubular and actinomorphic^17,26,36^. Individuals characterized by a slightly bilabiate calyx were considered to be of hybrid origin. In total, 15 samples were identified as common sage, 34 as Greek sage and 26 as their putative hybrid.

In defining the sampling area, the main criteria were that all samples must be (1) within a pollinator’s range and (2) strictly from the area of sympatry. Because the expected foraging distances for honey bees is greater than 1 km^74–76^, up to few hundred metres for solitary bees^77,78^ and at least 250 m for bumble bees^79,80^, all sampled specimens were obtained within a single pollinator’s range (i.e., the greatest distance between two sampled individuals was no greater than 1 km). To meet the second criterion, three groups (i.e., parental species and hybrids) were sampled in unequal proportions and in limited numbers. Since studied species do not naturally reproduce clonally and are characterized by distinctive bush-like habit, there was no risk of sampling genetically identical individuals. All of the samples were collected from neglected and abandoned vineyards and olive groves surrounding a local settlement, on a site of S-SW exposition, at the altitude ranging from 30 to 110 m. The majority of observed individuals was grouped in a large stand and a typical distance between sampled individuals ranged from 5 to 10 meters. The sampling was performed in a way that the entire stand was equally sampled.

### Laboratory methods

Based on a previous taxonomic revision of the section *Salvia*^36^, 23 qualitative and 16 quantitative traits were selected and measured from digitized photographs using a Dino-Lite pro Digital Microscope and DinoCapture 2.0 software (AnMo Electronics Corp, New Taipei City, Taiwan).

Total genomic DNA was extracted from silica-gel-dried leaf tissue using a GenElute Plant Genomic DNA Miniprep Kit (Sigma-Aldrich, St. Louis, Missouri, USA). The DNA concentrations were measured using a P330 Nanophotometer (IMPLEN, Munich, Germany).

For fine-scale analysis of the population genetic structure of the sampled groups, seven microsatellite loci (SoUZ003, SoUZ006, SoUZ007, SoUZ009, SoUZ013, SoUZ014 and SoUZ020) that were previously characterized in common sage and successfully cross-amplified in Greek sage^81–83^ were amplified. Details on the polymerase chain reaction (PCR) conditions and cycle profiles may be found in Radosavljević *et al*.^62^. Polymerase chain reaction products were analysed by capillary electrophoresis on a ABI 3730xL DNA analyser (Applied Biosystems, Foster City, CA, USA) provided by Macrogen DNA service (Seoul, Korea), and alleles were scored using GeneMapper 4.0 (Applied Biosystems).

For a genome-wide assessment of the genetic diversity, the AFLP technique was used based on the protocol of Carović-Stanko *et al*.^84^ with some modifications. For selective amplification, four primer combinations were used as follows: VIC-EcoRI-ACG + Tru1I-CGA, NED-EcoRI-AGA + Tru1I-CAC, FAM-EcoRI-ACA + Tru1I-CAC and PET-EcoRI-ACC + Tru1I-CGA. Amplified PCR products were analysed using an ABI 3730xL analyser (Applied Biosystems), and the GeneMapper 4.0 software (Applied Biosystems) was used for final allele scoring. Reactions of genomic DNA restriction, adapter ligation, and preselective and selective amplification were performed using a GeneAmp PCR System 9700 (Applied Biosystems, Foster City, CA, USA). The selection of markers for further statistical analysis and the estimation of the error rate per primer combination were performed as suggested by Herrmann *et al*.^85^.

To assess the level of epigenetic variability of the studied groups, MSAP analysis was applied in accordance with Nuskern *et al*.^86^. Analysis and scoring of the amplified PCR products were the same as in the AFLP analysis.

### Morphometric data analysis

Shannon’s information index as a measure of qualitative trait diversity was calculated for each trait according to Lewontin^87^ (1972) as well as separately for each species. Repeated measure analysis of variance was performed using PROC GLM in SAS^88^ with species membership as categorical independent variable and Shannon’s information index of each qualitative trait as response variable. *Post hoc* Bonferroni’s adjustments were used to compare the mean values of Shannon’s information index between species at a significance level of P < 0.05. Univariate analysis of variance was performed using PROC GLM in SAS^88^ to test for differences in mean values between species (categorical independent variable) for 19 quantitative morphological traits (response variables). *Post hoc* comparisons of the means were performed using Tukey’s Studentized Range test at P < 0.05.

To combine the information from the qualitative and quantitative traits into a single distance measure, Gower’s distance^89^ was calculated between all pairs of individuals with the algorithm implemented in PAST ver. 2.01^90^. Pairwise Gower’s distance matrices among individuals were calculated separately based on qualitative and quantitative data and compared by calculating the correlation coefficients and performing Mantel’s test^91^. The randomization procedure as implemented in NTSYS-pc^92^ included 1,000 permutations. To test the significance of the differences in mean intraspecies Gower’s distances among the species, Kruskal-Wallis (among all species) and Wilcoxon rank-sum (between all pairs of species) non-parametric tests were performed using SAS Release 9.2^88^. A neighbour-net diagram, which is well suited to depict reticulate relationships^93^, was constructed from Gower’s distances using SplitsTree 4^94^.

### SSR data analysis

GENEPOP 4.0^95^ was used to estimate population genetic parameters (the average number of alleles per locus, *N*_*av*_; the observed heterozygosity, *H*_*O*_; the expected heterozygosity, *H*_*E*_; and the inbreeding coefficient, *F*_*IS*_) and to test genotypic frequencies across all loci for conformance to Hardy-Weinberg (HW) expectations. As a measure of the number of alleles per locus is independent of sample size, allelic richness (*N*_*ar*_) was calculated using the FSTAT v.2.9.3.2 package^96,97^, and the number of private alleles (*N*_*pr*_) per species was assessed using MICROSAT^98^. The estimates of *N*_*ar*_, *H*_*O*_ and *H*_*E*_ in each species were compared by repeated measure analysis of variance followed by *post hoc* Bonferroni’s adjustments using PROC GLM in SAS^88^. The species membership was used as categorical independent variable and the estimates of *N*_*ar*_, *H*_*O*_ and *H*_*E*_ as response variables. The private allelic richness (*N*_*par*_) for each species was calculated by averaging the number of private alleles across possible subsets of sampled chromosomes while adjusting for differences in sample size across species. The calculations were performed using the rarefaction procedure as implemented in ADZE 1.0^99^.

Genetic distances between pairs of samples were calculated using the proportion-of-shared-alleles distances (*D*_*psa*_)^100^ as implemented in MICROSAT^98^. A neighbour-net diagram was produced as described previously. Bootstrap support was obtained using 1,000 replicates generated by MICROSAT and subsequently used in the cluster analysis performed by the neighbor-joining method as implemented in the NEIGHBOR and CONSENSE programs in the PHYLIP ver. 3.6b software package^101^. The significance of the differences in mean intraspecies distances among species was assessed as previously described.

Analysis of molecular variance^102^ (AMOVA) was used to partition the total genetic variance into within and among species in Arlequin ver. 2.000^103^. Pairwise comparisons examined with analysis of molecular variance resulted in values of *ϕ*_*ST*_ that are equivalent to the proportion of the total variance that is partitioned between the two species.

A model-based clustering method was applied to infer the genetic structure and define the number of clusters using STRUCTURE ver. 2.3.3^104^. Given a value for the number of clusters, this method assigns individual genotypes from the entire sample to clusters so that linkage disequilibrium (LD) was maximally explained. Thirty runs for each cluster (*K*) ranging from one to 11 were performed on the Isabella computer cluster at the University of Zagreb (Croatia), University Computing Centre (SRCE). Each run consisted of a burn-in period of 200,000 steps, followed by 10^6^ Monte Carlo Markov Chain (MCMC) replicates assuming an admixture model and correlated allele frequencies. The choice of the most likely number of clusters (*K*) was performed by comparing the average estimates of the likelihood of the data, *ln[Pr(X|K)]*, for each value of *K*, as well as by calculating an ad hoc statistic Δ*K* based on the rate of change in the log probability of data between successive *K* values, as described by Evanno *et al*.^105^ and implemented in STRUCTURE HARVESTER v0.6.92^106^. Runs were clustered and averaged using CLUMPAK^107^.

The Bayesian method implemented by NewHybrids 1.1^108^ was used to assign individuals to one of six classes: two pure (parental *S*. *officinalis* and *S*. *fruticosa*) and four putative (F_1_, F_2_, and backcrosses with the parental populations) hybrids. The program ran without any prior information regarding the putative hybrid status of collected individuals and with the uninformative Joffrey’s prior option for both mixing proportions and allele frequencies. The results were based on the average of five independent runs consisting of a burn-in phase of 100,000 steps and 500,000 MCMC sweeps. Following the suggestions of Anderson and Thompson^108^, individual genetic assignment to classes was based on a minimum posterior probability threshold (*Tq*) of 0.50.

### AFLP data analysis

AFLP-amplified fragments were scored for the presence (1) or absence (0) of homologous bands to create binary matrices. The AFLP diversity within species was estimated by determining the percentage of polymorphic bands (%P) and Shannon’s information index. The mean values of Shannon’s information index among species were tested as previously described.

Pairwise genetic distances were calculated using Dice’s coefficient^109^ (Dice, 1945), and a neighbour-net diagram was constructed as previously described. Bootstrap support was obtained using 1,000 samples in the cluster analysis performed by the neighbour-joining method as implemented in TREECON for Windows version 1.3 b^110^. The significance of the differences in mean intraspecies distances among species was assessed as previously described. Analysis of molecular variance and the analyses in Structure and NewHybrids were performed using the same parameters as previously described.

### MSAP data analysis

In the MSAP analyses, after the *Eco*RI/*Hpa*II and *Eco*RI/*Msp*I reactions, four distinct profiles were obtained as follows: (I) fragments present in both profiles (1/1), unmethylated state; (II) fragments present only in the *Eco*RI/*Msp*I profiles (0/1), hemi- or full-methylation of internal cytosine (^HMe^CG or ^Me^CG-sites); (III) fragments present only in *Eco*RI/*Hpa*II profiles (1/0), hemi-methylation of external cytosine (^HMe^CCG-sites); and (IV) fragments absent in both profiles (0/0), uninformative state. A ‘*Mixed-Scoring 2*’ approach as implemented in R script MSAP_calc.r^111^ was used to generate a matrix of binary data for each of the three classes of markers corresponding to three subepiloci: *u*-subepilocus (coding of unmethylated sites), *m*-subepilocus (coding of ^Hme^CG or ^Me^CG-sites), and *h*-subepilocus (coding of ^HMe^CCG-sites). Further analyses were performed in the same manner as in the case of the AFLP data.

### Environmental-niche modelling

To test the presumption that the central Adriatic region is well suited for survival of common sage, but not Greek sage, environmental-niche modelling for both species was independently performed using MAXENT ver. 3.3.3k.^112^. The analysis was based on 90 occurrence records for common sage and 70 occurrence records for Greek sage. From the WorldClim dataset, 19 bioclimatic variables for the present time and at a resolution of 30 arc-seconds were downloaded^113^. To test the multicollinearity among the bioclimatic variables for each species separately, “usdm” R package^114^ was used to calculate variance inflation factors (VIF) and only the bioclimatic variables with VIF < 10 were used for further analysis. For a selection of an appropriate value of Maxent’s regularization multipliers for obtained sets of bioclimatic variables, ENMTools was used^115^. After the environmental suitability model was constructed, ENM residuals (calculated for each occurrence record as 1 – Y, where Y was ENM result) were used to construct Moran’s I correlogram (i.e. to calculate the spatial autocorrelation factors (Moran’s I) for multiple distance classes) as implemented in SAM v4.0 software^116^. Obtained environmental suitability models were visualized in ArcGIS ver. 10.1. (Esri, Redlands, CA, USA).

## Supplementary information

Supplementary Information

## Data Availability

The datasets used and/or analysed during the current study are available from the corresponding author upon request.
