# Supplementary material for: Morphological, genetic and epigenetic aspects of homoploid hybridization between Salvia officinalis L. and Salvia fruticosa Mill
Source: Sci Rep. 2019 Mar 1;9:3276. doi: 10.1038/s41598-019-40080-0 (PMC6397195; doi:10.1038/s41598-019-40080-0)

Morphological, genetic and epigenetic aspects of homoploid hybridization between *Salvia officinalis* L. and *Salvia fruticosa* Mill.

Ivan Radosavljević[1,2], Sandro Bogdanović[2,3], Ferhat Celep[4], Maja Filipović[1], Zlatko Satovic[2,5], Boštjan Surina[6,7], Zlatko Liber[1,2*]

[1]University of Zagreb, Faculty of Science, Department of Biology, Division of Botany, Marulićev trg 9A, HR 10000 Zagreb, Croatia.
[2]Centre of Excellence for Biodiversity and Molecular Plant Breeding (CroP-BioDiv), Svetošimunska cesta 25, HR 10000 Zagreb, Croatia.
[3]University of Zagreb, Faculty of Agriculture, Department of Agricultural Botany, Svetošimunska cesta 25, HR 10000 Zagreb, Croatia.
[4] Department of Biology, Faculty of Arts and Sciences, Kırıkkale University, Kırıkkale, Turkey
[5]University of Zagreb, Faculty of Agriculture, Department of Seed Science and Technology, Svetošimunska cesta 25, HR 10000 Zagreb, Croatia,
[6]Natural History Museum Rijeka, Lorenzov prolaz 1, HR 51000 Rijeka, Croatia,
[7]University of Primorska, Faculty of Mathematics, Natural Sciences and Information Technologies, Glagoljaška 8, SI 6000 Koper, Slovenia

*zlatko.liber@biol.pmf.hr

Supplementary Table S1. Proportion of plants exhibiting a certain trait (*p*) and Shannon's diversity index (*H*) of 23 qualitative morphological traits.

No.	Organ	Trait	Abbr.	*S. officinalis*		Hybrids		*S. fruticosa*		Overall	
				p	*H*	*p*	*H*	*p*	*H*	*p*	*H*
1	Inflorescence	Occurance of bracts in the first lower verticillaster	BLV	0.875	0.544	0.720	0.855	0.765	0.787	0.773	0.772
2	Inflorescence	Occurance of bracts in the second and upper verticillasters	BUV	0.500	1.000	0.600	0.971	0.765	0.787	0.653	0.931
3	Inflorescence	Occurance of leaves in the zone of inflorescence	LI	0.063	0.337	0.640	0.943	0.765	0.787	0.573	0.984
4	Inflorescence axis	Trichome type: 1 Eglandular 1.1 Patent	IEP	0.063	0.337	0.360	0.943	0.588	0.977	0.400	0.971
5	Inflorescence axis	Trichome type: 1 Eglandular 1.2 Subadpressed	IEA	1.000	0.000	1.000	0.000	1.000	0.000	1.000	0.000
6	Inflorescence axis	Trichome type: 2 Glandular 2.1 Short glandular	IGS	0.000	0.000	0.000	0.000	0.765	0.787	0.347	0.931
7	Inflorescence axis	Trichome type: 2 Glandular 2.2 Long capitulate	IGL	0.000	0.000	0.000	0.000	0.412	0.977	0.187	0.694
8	Inflorescence axis	Trichome type: 2 Glandular 2.3 Sessile	IGSS	1.000	0.000	1.000	0.000	0.971	0.191	0.987	0.102
9	Inflorescence axis	Trichome type: 2 Glandular 2.4 Stalked	IGST	0.000	0.000	0.000	0.000	0.176	0.672	0.080	0.402
10	Inflorescence branches	Occurance of secondary branches	OSB	0.500	1.000	0.680	0.904	0.824	0.672	0.707	0.873
11	Floral bract	Trichome type: 1 Eglandular 1.1 Patent	BEP	0.063	0.337	0.600	0.971	0.971	0.191	0.653	0.931
12	Floral bract	Trichome type: 1 Eglandular 1.2 Subadpressed	BEA	1.000	0.000	0.880	0.529	0.706	0.874	0.827	0.665
13	Floral bract	Trichome type: 2 Glandular 2.1 Short glandular	BGS	0.000	0.000	0.120	0.529	0.676	0.908	0.347	0.931
14	Floral bract	Trichome type: 2 Glandular 2.2 Long capitulate	BGL	0.000	0.000	0.040	0.242	0.441	0.990	0.213	0.748
15	Floral bract	Trichome type: 2 Glandular 2.3 Sessile	BGSS	1.000	0.000	0.960	0.242	0.971	0.191	0.973	0.177
16	Floral bract	Trichome type: 2 Glandular 2.4 Stalked	BGST	0.000	0.000	0.040	0.242	0.118	0.523	0.067	0.353
17	Flower pedicel	Trichome type: 1 Eglandular 1.1 Patent	FEP	0.000	0.000	0.480	0.999	0.794	0.734	0.520	0.999
18	Flower pedicel	Trichome type: 1 Eglandular 1.2 Subadpressed	FEA	1.000	0.000	0.600	0.971	0.353	0.937	0.573	0.984
19	Flower pedicel	Trichome type: 2 Glandular 2.1 Short glandular	FGS	0.000	0.000	0.000	0.000	0.118	0.523	0.053	0.300
20	Flower pedicel	Trichome type: 2 Glandular 2.2 Long capitulate	FGL	0.000	0.000	0.000	0.000	0.176	0.672	0.080	0.402
21	Flower pedicel	Trichome type: 2 Glandular 2.3 Sessile	FGSS	1.000	0.000	1.000	0.000	0.676	0.908	0.853	0.601
22	Flower pedicel	Trichome type: 2 Glandular 2.4 Stalked	FGST	0.000	0.000	0.000	0.000	0.412	0.977	0.187	0.694
23	Calyx	Calyx appendices	CAP	1.000	0.000	0.960	0.242	0.176	0.672	0.613	0.963
	Mean[1]				0.155[c]		0.417[b]		0.684[a]		0.670
	N_{mono}[2]				17		9		1		1

[1]Means followed by different letters are significantly different at $P < 0.05$ after Bonferroni adjustment, [2]Number of monomorphic ($H = 0.000$) qualitative traits

Supplementary Table S2. Frequencies of specific trichome patterns in *S. officinalis, S. fruticosa* and their hybrids.

No.	Trichome pattern	Number of plants			
		S. officinalis	Hybrids	*S. fruticosa*	Overall
1	00010/010010/010010	14	5		19
2	10010/110010/010010	1	3		4
3	00010/110010/010010		5	1	6
4	10010/110010/100010		4		4
5	00010/010010/100010		3		3
6	00010/001101/100010		1		1
7	00010/011010/010010		1		1
8	00010/100010/100010		1		1
9	00010/110010/110010		1		1
10	10010/010010/110010		1		1
11	10010/101010/100010		1		1
12	01010/111110/100010			2	2
13	11010/101010/100010			2	2
14	00010/110010/100001			1	1
15	00010/111010/010000			1	1
16	00010/111111/100001			1	1
17	00011/111011/010100			1	1
18	01000/000000/010010			1	1
19	01010/110010/110010			1	1
20	01010/111110/010010			1	1
21	01010/111110/100001			1	1
22	01110/101010/100010			1	1
23	01110/111010/011010			1	1
24	01110/111110/110010			1	1
25	10010/110010/100011			1	1
26	10010/110010/100101			1	1
27	10111/110110/100010			1	1
28	11010/101010/100011			1	1
29	11010/101110/100010			1	1
30	11010/110010/100001			1	1
31	11011/101011/100011			1	1
32	11011/110010/100001			1	1
33	11110/101010/100011			1	1
34	11110/101110/100001			1	1
35	11110/101110/100010			1	1
36	11110/111110/100000			1	1
37	11110/111110/100101			1	1
38	11110/111110/110010			1	1
39	11110/111110/111110			1	1
40	11110/111111/011110			1	1
41	11111/110010/100011			1	1
42	11111/110010/111111			1	1
	No. of individuals	15	26	34	75
	No. of patterns	2	11	32	42

*Digits 1 to 5 correspond to presence/absence of different trichome types on the inflorescence axis (qualitative traits nos. 4-9; excluding trait no. 5 that was monomorphic from Supplementary Table S1), 6 to 11 on the floral bracts (qualitative traits nos. 11-16 from Supplementary Table S1) and 12 to 17 on flower pedicel (qualitative traits 17-22 from Supplementary Table S1). Trichome patterns are ordered by the frequency of the occurrence in a species.

Supplementary Table S3. Mean values and range of 16 measured quantitative morphological traits.

No.	Organ	Trait (unit)	N[3]	Abbr.	P(F)[1]	*S. officinalis*			Hybrids			*S. fruticosa*		
						Mean[2]		Range	Mean		Range	Mean		Range
1	Inflorescence	Inflorescence length (cm)	1	IL	***	11.75	b	6.0-21.0	23.48	a	9.7-47.5	27.83	a	9.7-55.3
2	Inflorescence	Number of internodes on inflorescence	1	NII	***	6.07	c	4.0-8.0	9.62	b	7.0-14.0	11.35	a	5.0-19.0
3	Inflorescence	Number of flowers in lower verticillaster	1	NFV	*	9.87	b	4.0-26.0	16.46	a	6.0-41.0	17.85	a	5.0-44.0
4	Inflorescence branches	Number of primary branches	1	NPB	**	0.93	b	0.0-2.0	2.43	ab	0.0-8.0	3.82	a	0.0-12.0
5	Floral bract	Bract length (mm)	1	BL	*	15.33	b	12.3-19.4	18.26	a	2.9-30	15.62	ab	7.3-24.9
6	Floral bract	Bract width (mm)	1	BW	***	9.36	a	6.1-12.5	8.20	a	1.1-14.2	6.42	b	3.7-9.1
7	Calyx	Trichome type: 1 Eglandular 1.1 Patent	5	CEP	***	0.00	c	0.0-0.0	0.56	b	0.0-1.0	0.99	a	0.6-1.0
8	Calyx	Trichome type: 1 Eglandular 1.2 Subadpressed	5	CEA	***	1.00	a	1.0-1.0	0.66	b	0.0-1.0	0.11	c	0.0-1.0
9	Calyx	Trichome type: 2 Glandular 2.1 Short glandular	5	CGS	***	0.00	b	0.0-0.0	0.71	a	0.0-1.0	0.89	a	0.0-1.0
10	Calyx	Trichome type: 2 Glandular 2.2 Long capitulate	5	CGL	***	0.00	c	0.0-0.0	0.22	b	0.0-1.0	0.92	a	0.0-1.0
11	Calyx	Trichome type: 2 Glandular 2.3 Sessile	5	CGSS	***	1.00	a	1.0-1.0	0.96	a	0.0-1.0	0.31	b	0.0-1.0
12	Calyx	Trichome type: 2 Glandular 2.4 Stalked	5	CGST	***	0.31	c	0.0-1.0	0.74	b	0.0-1.0	0.99	a	0.6-1.0
13	Calyx	Calyx length (mm)	5	CL	***	13.45	a	11.9-15.3	11.83	b	8.5-15	9.32	c	8.1-10.6
14	Calyx	Calyx lobes length (min) (mm)	5	CLMI	***	1.27	c	0.6-2.4	2.54	a	1.4-4.2	1.69	b	1.2-2.5
15	Calyx	Calyx lobes length (max) (mm)	5	CLMX	***	5.10	a	3.6-6.5	4.06	b	2.3-5.6	2.29	c	1.6-3.3
16	Calyx	Calyx nervature: reticulate	5	CNR	***	0.87	a	0.0-1.0	0.68	a	0.0-1.0	0.25	b	0.0-1.0

[1]P(F), significance of ANOVA's F-test: *** significant at $P < 0.001$, ** significant at $0.001 < P < 0.01$, * significant at $0.01 < P < 0.05$,. ns depicts non-significant values ($P > 0.05$)

[2]Means in the same row followed by different letters are significantly different at $P < 0.05$ based on Tukey's test

[3]Number of measurements per individual

Supplementary Table S4. Microsatellite diversity of *S. officinalis*, *S. fruticosa* and their hybrids.

Species	n	N_{av}	N_{ar}	N_{pr}	N_{par}	H_O	H_E	F_{IS}
S. officinalis	15	6.86	6.86[a]	10	2.00	0.610[ab]	0.749[a]	0.187***
Hybrids	26	7.43	6.48[a]	5	0.52	0.778[a]	0.678[a]	-0.147
S. fruticosa	34	3.43	3.00[b]	0	0.13	0.289[b]	0.333[b]	0.132**
P			0.016			0.004	0.007	

n - sample size; N_{av} - average number of alleles; N_{ar} - allelic richness; N_{pr} - total number of private alleles; N_{par} - private allelic richness; H_O - observed heterozygosity; H_E - expected heterozygosity; F_{IS} - inbreeding coefficient (Probabilities of heterozygote deficiency: "***" corresponds to significance at the 0.1% nominal level; "**" significance at the 1% nominal level; "*" significance at the 5% nominal level); *P* - *P*-value of the test for difference among species (Means followed by different letters are significantly different at $P < 0.05$ after Bonferroni adjustment.).

Supplementary Table S5. Interspecies ϕ_{ST} values and mean distance based on SSR, AFLP and MSAP analyses.

Analysis	Species	*S. officinalis*	Hybrids	*S. fruticosa*
SSR	*S. officinalis*	**0.684**	0.771	0.876
	Hybrids	0.144	**0.522**	0.519
	S. fruticosa	0.431	0.115	**0.289**
AFLP	*S. officinalis*	**0.297**	0.547	0.836
	Hybrids	0.460	**0.281**	0.421
	S. fruticosa	0.733	0.465	**0.178**
MSAP	*S. officinalis*	**0.508**	0.614	0.717
	Hybrids	0.146	**0.540**	0.565
	S. fruticosa	0.322	0.108	**0.470**

Interspecies ϕ_{ST} values are shown below the diagonal and mean distance among species based on SSR, AFLP and MSAP markers are shown above diagonal (proportion-of-shared alleles distance for SSRs and Dice's distance for AFLPs and MSAPs). Mean distances are provided along the diagonal (in bold). All ϕ_{ST} values are significant at $P < 0.0001$.

Supplementary Table S6. AMOVA for the partitioning of SSR, AFLP and MSAP diversity among and within taxa

Analysis	Source of variation	df	Variance components	% Total variance	ϕ-statistics	$P(\phi)$
SSR	Among species	2	0.532	22.57	0.226	<0.0001
	Within species	147	1.824	77.43		
AFLP	Among species	2	14.769	57.19	0.572	<0.0001
	Within species	72	11.055	42.81		
MSAP	Among species	2	22.419	18.66	0.187	<0.0001
	Within species	72	97.754	81.34		

$P(\phi)$ - ϕ-statistical probability level after 10,000 permutations

Supplementary Table S7. Genetic and epigenetic diversity of studied groups as revealed by AFLP and MSAP analyses

Analysis	Species	*n*	*%P*	*H*
AFLPs	*S. officinalis*	15	0.455	0.304[b]
	Hybrids	26	0.739	0.447[a]
	S. fruticosa	34	0.582	0.322[b]
	P			0.002
MSAPs	*S. officinalis*	15	0.545	0.325[b]
	Hybrids	26	0.731	0.367[a]
	S. fruticosa	34	0.715	0.328[b]
	P			0.001

n - sample size; *%P* - proportion of polymorphic loci; *H* - Shannon's information index; *P* - *P*-value of the test for difference among species (Means followed by different letters are significantly different at $P < 0.05$ after Bonferroni adjustment.).

Supplementary Table S8. Relative contributions of bioclimatic variables to environmental niche models for *S. officinalis* and *S. fruticosa*

		% CONTRIBUTION	
	BIOCLIMATIC VARIABLES	*S. officinalis*	*S. fruticosa*
BIO1	Annual Mean Temperature	*n/a*	*n/a*
BIO2	Mean Diurnal Range (Mean of monthly (max temp - min temp))	*n/a*	*n/a*
BIO3	Isothermality ((BIO2/BIO7)x100)	0.2	0.6
BIO4	Temperature Seasonality (standard deviation *100)	10.4	28.9
BIO5	Max Temperature of Warmest Month	*n/a*	*n/a*
BIO6	Min Temperature of Coldest Month	*n/a*	*n/a*
BIO7	Temperature Annual Range (BIO5-BIO6)	*n/a*	*n/a*
BIO8	Mean Temperature of Wettest Quarter	2.3	0.2
BIO9	Mean Temperature of Driest Quarter	24.2	8.8
BIO10	Mean Temperature of Warmest Quarter	1.9	*n/a*
BIO11	Mean Temperature of Coldest Quarter	*n/a*	*n/a*
BIO12	Annual Precipitation	*n/a*	23.5
BIO13	Precipitation of Wettest Month	33.3	*n/a*
BIO14	Precipitation of Driest Month	27.7	*n/a*
BIO15	Precipitation Seasonality (Coefficient of Variation)	*n/a*	37.9
BIO16	Precipitation of Wettest Quarter	*n/a*	*n/a*
BIO17	Precipitation of Driest Quarter	*n/a*	*n/a*
BIO18	Precipitation of Warmest Quarter	*n/a*	*n/a*
BIO19	Precipitation of Coldest Quarter	*n/a*	*n/a*

n/a – bioclimatic variables that were not used for species model construction

Supplementary Figure S1. Venn diagram illustrating the number of shared and private microsatellite alleles in the studied taxa.

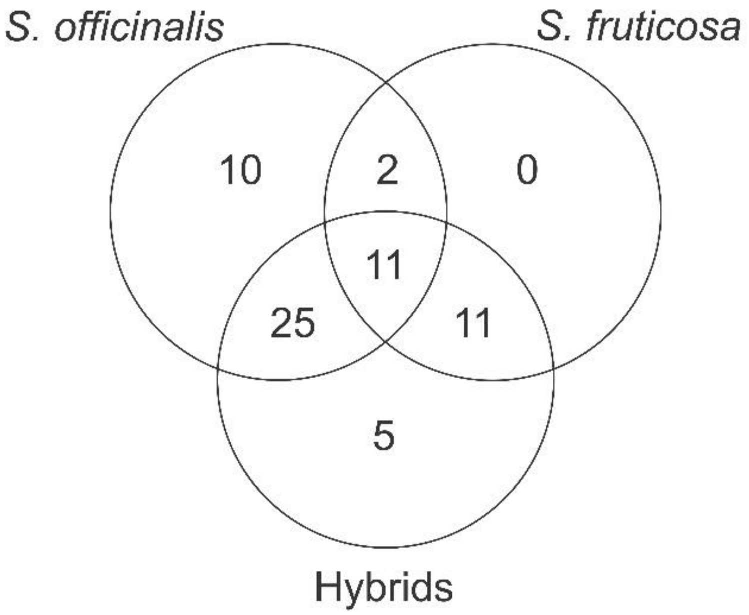

Supplementary Figure S2. Calyx shapes and trichome types in *S. officinalis* (1), hybrids (2) and *S. fruticosa* (3). The upper scale bar refers to trichomes, the lower to calyces. All scales are approximate and for illustration only.

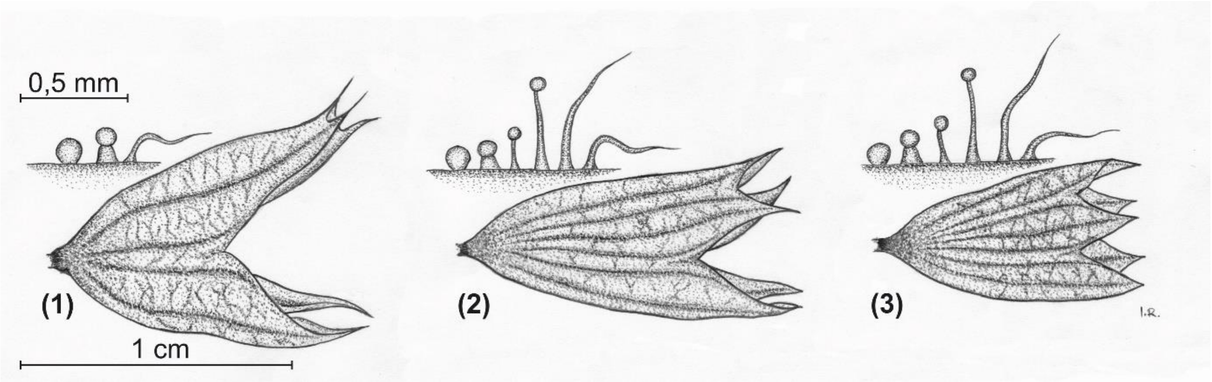

Supplement: Supplementary file 1 — Supplementary Information [file 41598_2019_40080_MOESM1_ESM.pdf]
